# 3R and 4R tau isoforms in paired helical filaments in Alzheimer’s disease

**DOI:** 10.1007/s00401-013-1191-9

**Published:** 2013-11-09

**Authors:** Masato Hasegawa, Sayuri Watanabe, Hiromi Kondo, Haruhiko Akiyama, David M. A. Mann, Yuko Saito, Shigeo Murayama

**Affiliations:** 1 Department of Neuropathology and Cell Biology, Tokyo Metropolitan Institute of Medical Science, Setagaya-ku, Tokyo, 156-8506 Japan; 2 Histology Center, Tokyo Metropolitan Institute of Medical Science, Setagaya-ku, Tokyo, 156-8506 Japan; 3 Dementia Research Project, Tokyo Metropolitan Institute of Medical Science, Setagaya-ku, Tokyo, 156-8506 Japan; 4 Centre for Clinical and Cognitive Neuroscience, Institute of Brain Behavior and Mental Health, University of Manchester, Salford, M6 8HD UK; 5 Department of Laboratory Medicine, National Center Hospital, NCNP, 4-1-1 Ogawahigashi, Kodaira, Tokyo 187-8502 Japan; 6 Department of Neuropathology, Tokyo Metropolitan Institute of Gerontology, Itabashi-ku, Tokyo, 173-0015 Japan

Isoform-specific tau antibodies RD3 and RD4 are useful tools for investigating expression and localization of three-repeat (3R) and four-repeat (4R) tau isoforms. Recently, transition from 3R to 4R tau in Alzheimer’s disease (AD) was proposed based on immunohistochemical studies with RD3 and RD4 [3]. Here, we show that two factors influence immunoreactivity to these antibodies. First, deamidation at the RD4 epitope abrogates immunoreactivity to RD4, and second, presentation of RD3 and RD4 epitopes is reciprocally affected by protease. Asparagine at position 279 in the RD4 epitope is predominantly deamidated to aspartic acid in pathological tau in AD brains [2, 4]. Consequently, the presence of 4R tau in AD pathologies may be underestimated when RD4 is used. However, anti-4R (available from Cosmo Bio Co., Ltd.) raised against RD4 peptide with N279D substitution stained both wild-type and deamidated 4R tau, and strongly stained RD3+/RD4− tangles and smearing tau fragments in Sarkosyl-insoluble fraction of AD brain [2].

**Fig. 1:**
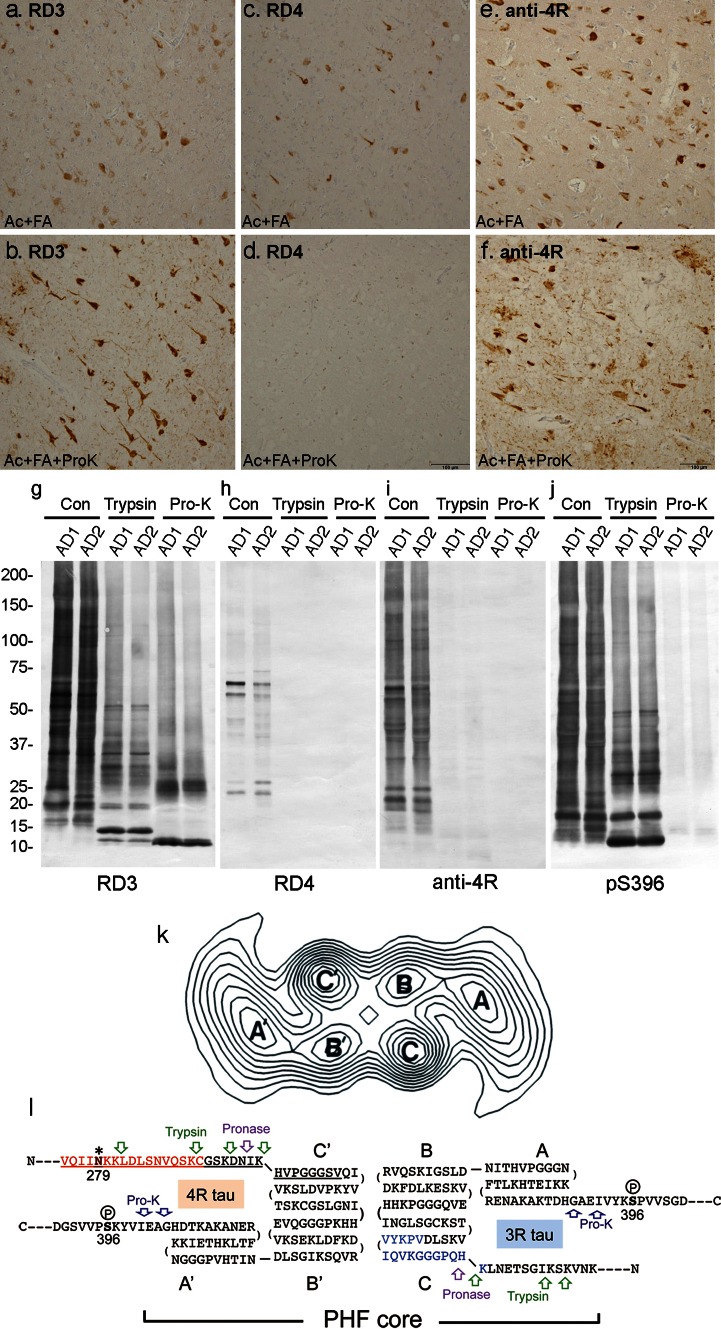
**a**–**f** Immunostaining of AD sections after Ac and FA treatment before (**a**, **c**, **e**) and after (**b**, **d**, **f**) Pro-K treatment, using RD3 (**a**, **b**), RD4 (**c**, **d**) and anti-4R (**e**, **f**). *Bar* 100 μm. **g**–**j** Immunoblots of Sarkosyl-insoluble tau from two AD brains, before (*Con*) and after treatments with trypsin or Pro-K, using RD3 (**g**), RD4 (**h**), anti-4R (**i**) and pS396 (**j**). **k**–**l** Computed cross-section through a paired helical filament (**k**) [reproduced from Ref. [1], with permission of the publisher], a predicted folding model of 3R and 4R tau in PHF (**l**). RD3 and RD4 epitopes are indicated by *blue* and *red*, respectively. 4R tau specific insertion is indicated by *underlining*. The deamidation site N279 is indicated by *asterisks*. Phosphorylation of Ser396 is indicated. Possible trypsin, pronase and Pro-K cleavage sites are indicated in *green*, *purple* and *dark blue arrows*, respectively. The protease-resistant domain of PHF is indicated as *PHF-core*

It was reported that RD3 stained abundant ghost tangles in entorhinal cortex and tangles in CA1, but failed to stain fine processes of tangles and threads [3], while RD4 failed to detect ghost tangles in entorhinal cortex [3]. To understand these findings, we examined the influence of protease on immunoreactivity. Paraffin sections of AD brains were treated with 10 μg/mL Proteinase K (Pro-K) for 30 min after autoclaving (Ac) and formic acid (FA) treatment. RD3 staining was strongly enhanced (Fig. 1a, b). Conversely, RD4 immunoreactivity almost completely disappeared after Pro-K treatment (Fig. 1c, d). Not only ghost tangles but also RD3−/RD4+ tangles and their processes became RD3-positive after Pro-K treatment (Fig. 1a, b), strongly suggesting that the RD3 epitope was buried in tau filaments of intracellular tangles and threads, and was exposed by Pro-K treatment. Contrary to expectation, anti-4R staining was also enhanced by Pro-K treatment (Fig. 1e, f). It is possible that the recognition site of anti-4R is distinct from that of RD4 and is exposed by Pro-K treatment of sections. Anti-4R antibody may recognize the carboxyl-half of the antigen peptide, while RD4 recognizes the amino-terminal half around N279. Pro-K treatment was also effective in immunostaining of free-floating AD sections with a lower concentration.

To confirm these findings biochemically, Sarkosyl-insoluble fractions from two AD brains were treated with trypsin or Pro-K, then immunoblotted with RD3, RD4, anti-4R and anti-pS396 (Fig. 1g–j). RD3 strongly stained many bands and smears, as seen with pS396 (Fig. 1g, j), whereas RD4 only labeled the 64/68 kDa doublet and some fragments at ~25 kDa (Fig. 1h). Anti-4R strongly stained the smears and fragments (Fig. 1i), suggesting that tau in these RD4-negative anti-4R-positive bands and smears is deamidated at N279. The weak RD4 and strong anti-4R immunoreactivities were completely abolished after trypsin or Pro-K treatment (Fig. 1h, i). This result is inconsistent with the immunohistochemistry, but protease sensitivity is likely different in fixed tissues. In contrast, the RD3 epitope was retained in the fragments, and RD3 strongly reacted with the protease-resistant 10–25 kDa bands after trypsin or Pro-K treatment (Fig. 1g). pS396 epitope was removed by Pro-K but not trypsin, suggesting a location outside the PHF core. Trypsin may not cleave the KSP site because of phosphorylation of Ser396. These results demonstrate reciprocal effects of protease treatment on RD3 and RD4 epitopes, indicating that RD4 epitope in tau in AD is susceptible to proteases, while RD3 epitope is highly resistant.

These results are consistent with previous findings. Wischik et al. identified two types of amino acid sequences, QPGGGKVQIVYK… (3R tau) and IKXVPGG… (4R tau), in 12-kDa tau fragment comprising the pronase-resistant core of PHFs [6] (see Fig. 1k). We identified HQPGGG…(3R tau) and HVPGGG… (4R tau) in 7–15 kDa trypsin-resistant fragments of PHF-tau in AD brains [5]. In both cases, 3R and 4R tau isoforms were detected, but the 4R tau N-terminus lacked the RD4 epitope. Based on these observations and a computed cross-section of PHF (Fig. 1k) [1], we propose a schematic model of tau folding in PHF (Fig. 1l). Analysis of the cross-sectional density in the PHF core on electron micrographs indicates the presence of two C-shaped morphological units, which correspond to the two strands of PHF, each with three domains (Fig. 1k) [1]. The RD3 epitope is buried in the PHF core and is normally masked by the N- or C-terminal region of tau, but is exposed in ghost tangles and/or in PHFs attacked by proteases. The RD4 epitope, which is mostly deamidated in PHF, is located slightly outside the core, where it can be digested by proteases (Fig. 1l). This model can explain the epitope masking of RD3 and RD4 and the reciprocal effects of degradation or protease treatment on the immunoreactivities.

This study indicates that differential presentation of epitopes can occur as a result of folding and processing, even when the epitopes are located in close proximity. Tau in PHFs appears to be processed gradually by intracellular proteases and more extensively in extracellular space during AD progression. We suggest that changes in immunoreactivity to antibodies reflect aging of tau in tangles or PHFs, which are composed of both 3R and 4R tau isoforms. We also show that Pro-K treatment of sections after Ac and FA treatment is useful for unmasking buried epitopes.
